# Inhibition of bromodomain and extra-terminal proteins targets constitutively active NFκB and STAT signaling in lymphoma and influences the expression of the antiapoptotic proteins BCL2A1 and c-MYC

**DOI:** 10.1186/s12964-024-01782-9

**Published:** 2024-08-27

**Authors:** Nadja M. Pieper, Julia Schnell, Daniela Bruecher, Stefan Knapp, Meike Vogler

**Affiliations:** 1 Institute for Experimental Pediatric Hematology and Oncology, Goethe University Frankfurt, Komturstrasse 3a, 60528 Frankfurt, Germany; 2 Institute for Pharmaceutical Chemistry, Germany and Structural Genomics Consortium, Buchmann Institute for Life Sciences, Goethe-University Frankfurt, Max-von-Laue- Str. 9, Biozentrum, 60438 Frankfurt am Main, Germany; 3 German Cancer Consortium (DKTK) Partner Site Frankfurt/Mainz, a Partnership between 10 DKFZ and University Hospital Frankfurt, Frankfurt, Germany; 4 University Cancer Center Frankfurt (UCT), University Hospital Frankfurt, Goethe University Frankfurt, Frankfurt, Germany

**Keywords:** BETi, BCL2-proteins, BCL2A1, c-MYC, Epigenetics, Apoptosis, Lymphoma, PROTACs

## Abstract

The antiapoptotic protein BCL2A1 is highly, but very heterogeneously expressed in Diffuse Large B-cell Lymphoma (DLBCL). Particularly in the context of resistance to current therapies, BCL2A1 appears to play an important role in protecting cancer cells from the induction of cell death. Reducing BCL2A1 levels may have therapeutic potential, however, no specific inhibitor is currently available. In this study, we hypothesized that the signaling network regulated by epigenetic readers may regulate the transcription of BCL2A1 and hence that inhibition of Bromodomain and Extra-Terminal (BET) proteins may reduce BCL2A1 expression thus leading to cell death in DLBCL cell lines. We found that the mechanisms of action of acetyl-lysine competitive BET inhibitors are different from those of proteolysis targeting chimeras (PROTACs) that induce the degradation of BET proteins. Both classes of BETi reduced the expression of BCL2A1 which coincided with a marked downregulation of c-MYC. Mechanistically, BET inhibition attenuated the constitutively active canonical nuclear factor kappa-light-chain-enhancer of activated B-cells (NFκB) signaling pathway and inhibited p65 activation. Furthermore, signal transducer of activated transcription (STAT) signaling was reduced by inhibiting BET proteins, targeting another pathway that is often constitutively active in DLBCL. Both pathways were also inhibited by the IκB kinase inhibitor TPCA-1, resulting in decreased BCL2A1 and c-MYC expression. Taken together, our study highlights a novel complex regulatory network that links BET proteins to both NFκB and STAT survival signaling pathways controlling both BCL2A1 and c-MYC expression in DLBCL.

**Graphical Abstract:**

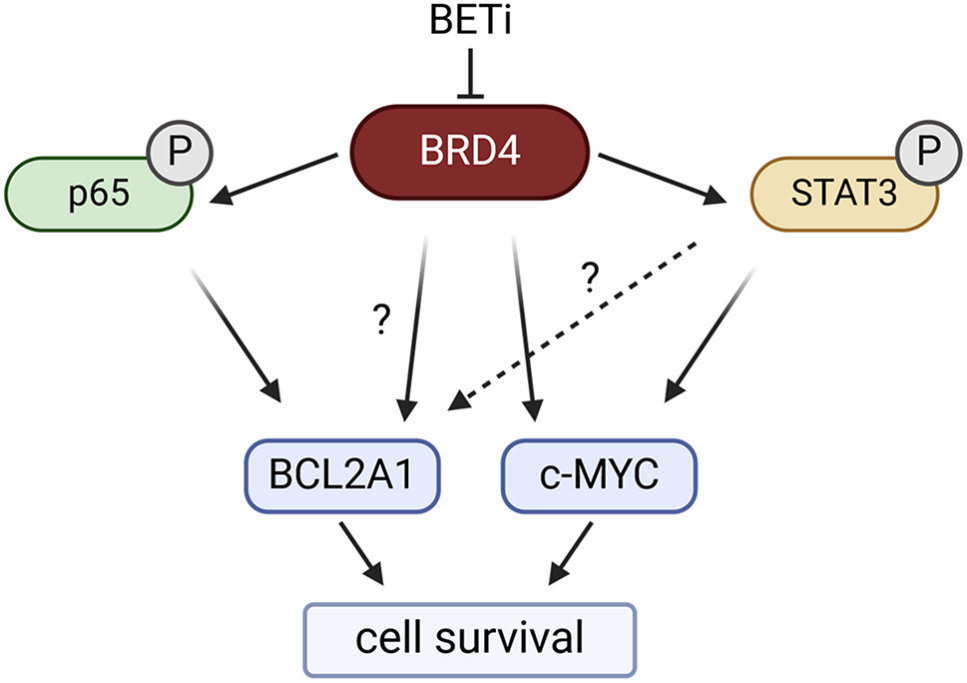

**Supplementary Information:**

The online version contains supplementary material available at 10.1186/s12964-024-01782-9.

## Introduction

DLBCL is a clinically aggressive form of Non-Hodgkin’s Lymphoma which can be classified into multiple subtypes, based on gene expression or mutational profiling [1–3]. The main routinely used subtypes are germinal center B-cell like (GCB) type and an activated B-cell-like (ABC) type, with the latter being associated with significantly worse disease progression and survival [1]. Genetic rearrangements and constitutively active B-cell receptor and NFκB signaling are common oncogenic drivers in the lymphomagenesis [2, 3]. The most frequent first line therapy is a combination of the chimeric monoclonal antibody rituximab that targets the B-lymphocyte antigen CD20 with the cytostatic agents cyclophosphamide, vincristine, doxorubicin and the corticosteroid prednisone (R-CHOP), which is successful in approximately 60% of all cases. Particularly in relapsed and refractory disease, targeted therapies have entered the clinic, and selective inhibition of anti-apoptotic proteins with concomitant sensitization to apoptosis represents an attractive treatment strategy [4]. However, the first approved BH3 mimetic, venetoclax, showed variable effectiveness in DLBCL patients and resistance was often observed [5]. One factor gaining importance in the context of resistance development against BH3 mimetics or chemotherapy is the anti-apoptotic protein BCL2A1/Bfl-1 [6–9].

BCL2A1 belongs to the anti-apoptotic members of the BCL2 protein family along with e.g. BCL2, BCLx_L_ and MCL-1. The anti-apoptotic proteins can be sequestered and neutralized by pro-apoptotic BH3-only proteins including BIM, NOXA or PUMA. Thus, cell fate is determined by the relative expression and binding of pro- and anti-apoptotic partners. Increased pro-apoptotic signals activate the effector proteins BAX and BAK that form pores in the outer mitochondrial membrane, leading to cytochrome c release, subsequent caspase activation and apoptosis induction [10]. While many solid cancers display upregulation of BCLx_L_ or MCL-1 compared to the respective healthy cells (reviewed in [11]), a basal upregulation of cellular BCL2A1 levels has been described in breast cancer, melanoma, or hematological and lymphoid cancers, such as DLBCL [9]. DLBCL displays a high but heterogeneous expression of BCL2A1 that is not associated with known DLBCL subtypes [12, 13].

Efforts to directly inhibit BCL2A1 have so far not yielded potent and selective inhibitors although progress has recently been made to develop a covalent inhibitor [14]. Studies in double-hit lymphoma suggest that an indirect downregulation of BCL2A1 levels can be achieved by targeting BET proteins [15]. Ongoing clinical trials evaluate the efficacy of BET inhibition in DLBCL patients in single drug and drug combination settings [16–18]. BET proteins bind to acetylated lysine residues acting as readers of the epigenetic code. They can modulate the expression of tissue specific gene sets through the formation of multiprotein complexes, e.g. through recruitment of the positive transcription elongation factor complex (p-TEFb), by interacting with superenhancers or by influencing the basal transcription of target genes [19, 20]. Inhibition of BET proteins can be achieved by classical competitive binding to the bromodomains and subsequent inhibition of the target molecule function [21]. Alternatively, selective PROTACs enable the degradation of the BET proteins of interest through the recruitment of E3 ubiquitin ligases to the target molecule, which induces ubiquitination resulting in proteasomal degradation [22]. It was previously reported that BET inhibition can lead to changes in the transcription of signaling molecules such as NFκB or molecules of the JAK/STAT signaling axis, as well as changes in the BCL2 protein family [23–25].

The family of NFκB proteins consists of RelA/p65, RelB, c-Rel, p50 and p52 that form cell-type and stimulus-dependent homo- or heterodimers. Activation of canonical NFκB signaling involves phosphorylation of inhibitors of IκB kinases (IKKs) in complex with NEMO which subsequently phosphorylate the NFκB Inhibitors (IκBs), most prominently IκBα. This leads to ubiquitination and proteasomal degradation of IκB proteins and the release of NFκB dimers, which translocate to the nucleus and influence the transcription of their target genes [26]. In the non-canonical pathway, activation of IKKs independently of NEMO leads to direct phosphorylation and partial proteolysis of the precursor protein p100, resulting in the p52 protein.

The interaction between NFkB activation and STAT signaling may be cell type- and context-dependent, with cooperative or antagonistic effects being reported [27, 28]. The family of STAT proteins regulates genes involved in proliferation, cell adhesion or immune evasion, among others. Especially, STAT3 signaling is often hyperactivated in cancer, including ABC DLBCL cells [29]. Here, cytokines such as IL-6 and IL-10, which are targets of NFκB, can induce STAT3 signaling in an autocrine manner [30]. Upon activation, phosphorylation of STAT3 triggers dimerization and nuclear translocation, resulting in the modulation of target gene transcription [31].

In this study, we asked whether BET inhibitors or degraders may be able to inhibit BCL2A1 expression in DLBCL. Given the complex epigenetic and post-transcriptional regulation of BCL2A1, we aimed to investigate the molecular mechanism by which BET inhibition may alter BCL2A1 expression and how this is associated with cell death induced by BET inhibition. Our study highlights that BET inhibition reduced both constitutively active canonical NFκB as well as STAT signaling, indicating that BCL2A1 expression is regulated by a complex network of survival signaling pathways in ABC and GCB DLBCL cell lines.

## Materials and methods

### Chemicals

The BET inhibitors JQ1, PLX50117, I-BET-151, ABBV-744, I-BET-726 and the BET degraders MZ1, dBET1, AT1 were kindly provided by the SGC (Goethe University, Frankfurt, Germany) and were dissolved in DMSO. In addition, dBET6 (Tocris, 6945), ABBV-075 (Mivebresib) (Selleckchem, S8400) and ARV-825 (Medchemexpress, HY-16954) were dissolved in DMSO and used at indicated concentrations. TPCA-1 (Sigma, T1452) was dissolved in DMSO and used at the indicated concentrations. The chemical structure and compound class of all BETi is provided in the supplementary methods.

### Cell culture

ABC cell lines SUDHL2, TMD8 and U2932 and GCB cell lines Pfeiffer and SUDHL6 were used in this study. SUDHL2 (RRID: CVCL_8795) and Pfeiffer (RRID: CVCL_3326) cells were obtained from American Type Culture Collection, SUDHL6 (RRID: CVCL_2206) and U2932 (RRID: CVCL_1896) were obtained from Deutsche Sammlung von Mikroorganismen und Zellkulturen (DSMZ) and TMD8 cells (RRID: CVCL_A442) were kindly provided by Martin Dyer (Leicester, UK). All cells were authenticated by STR profiling (DSMZ). Cells were cultured in RPMI-1640 GlutaMAX-I medium (Life Technologies) supplemented with 10% fetal calf serum (FCS) (Life Technologies) and 1% penicillin/streptavidin (P/S) (Life Technologies) at 37 °C in humidified CO_2_-enriched atmosphere for 2–3 month after thawing. Cells were routinely tested for mycoplasma contamination by PCR (Minerva-Biolabs). Unless otherwise indicated, cells were seeded at 2 × 10^5^ cells/ml for experiments.

### Analysis of viability and cell death

The viability of cells was determined by CellTiter-Glo^®^ Luminescent Cell Viability Assay (Promega) after 48 h of incubation with the indicated treatments. Luminescence was measured with the TECAN Infinfite^®^ 200 (Tecan) and the i-control software.

Cell death measurements were performed by FACS analysis. After 24–48 h incubation 100 µl of cell suspension were stained for apoptosis by AnnexinV/PI staining. In brief, 0.05 µl Annexin V-FITC (produced in house from pET21a vector and conjugated with FITC) and 5 µl PI (50 µg/ml in PBS) were added to 100 µl Annexin V buffer and mixed with the cell suspension. After 10 min incubation at RT cells were analyzed by Flow Cytometry using the FACS Canto II (BD Biosciences) and the Diva software. Living and dead cells were gated in FSC/SSC measurements. Cells were additionally gated into AnnexinV - /PI -, AnnexinV +/PI -, AnnexinV -/PI + and AnnexinV + /PI + cells, and only double negative cells were defined as viable.

### Western blotting

For Western blotting cells were seeded in 6 well plates and harvested after 24 h for endogenous expression or upon treatment for the indicated times. Cells were lysed for 25 min on ice with lysis buffer (1 M Tris HCl, 4 M NaCl, Triton X100, glycerol supplemented with protease inhibitor complex (PIC) (Roche) and freshly added 100nM PMSF, 1 M DTT, 100mM sodium-orthovanadate, 100mM β-glycerophosphate and 500mM sodium fluoride) and the protein concentration was determined using Pierce™ BCA Protein Assay Kit (Thermo Fisher Scientific). Samples were boiled with 6x loading dye, separated by SDS-PAGE and proteins were blotted onto Nitrocellulose membranes. Primary antibodies were incubated at 4 °C over night and for detection Pierce™ ECL Western Blot Solution (Thermo Fisher Scientific) was used. For detection of total protein after phospho-proteins the membranes were stripped by incubation in 0,4 M NaOH for 10 min. For immunoblotting the following antibodies were used:

Cell Signaling Technology: rabbit monoclonal anti-phospho-NF-κB p65 (Ser536) (93H1), Cat#3033, RRID: AB_331284; mouse monoclonal anti-phospho-IκBα (S32/S36) (5A5), Cat#9246; RRID: AB_2267145; rabbit polyclonal anti-IκBα, Cat#9242; RRID: AB_331623; rabbit polyclonal anti-NIK, Cat# 4994, RRID: AB_2297422; rabbit polyclonal anti-BIM Cat# 2819, RRID: AB_10692515, rabbit polyclonal anti-c-MYC Cat# 9402, RRID: AB_2151827; mouse monoclonal anti-STAT3 (124H6), Cat# 9139, RRID: AB_331757; rabbit monoclonal anti-phospho-STAT3 (Tyr705) (D3A7), Cat# 9145, RRID: AB_2491009; mouse monoclonal anti-phospho-STAT5 (Tyr694), Cat’9356, RRID: AB_331263; rabbit monoclonal anti-STAT5, Cat# 94,205, RRID: AB_2737403; Santa Cruz Biotechnology: mouse monoclonal NF-κB p65 (F-6), Cat#sc-8008, RRID: AB_628017; Millipore: mouse monoclonal anti-NFκB p52 Cat# 05-361, RRID: AB_309692; Abcam: rabbit monoclonal anti-BCLx_L_ (E18), Cat# ab32370, RRID: AB_725655; rabbit polyclonal anti-BCL2A1, Cat# ab75887, RRID: AB_1523197; Agilent: mouse monoclonal BCL2 (clone 124), Cat# M0887, RRID: AB_2064429; Enzo Life Sciences: rabbit polyclonal anti-MCL-1, Cat# ADI-AAP-240, RRID: AB_10997659; mouse monoclonal anti-NOXA (clone 114C307.1), Cat# ALX-804-408-C100, RRID: AB_2052079; Sigma-Aldrich: mouse monoclonal anti-Vinculin, Cat#V9131; RRID: AB_477629; Hytest: mouse monoclonal anti-GAPDH, Cat#5G4cc-6C5cc; RRID: AB_2858176; Bethyl: rabbit polyclonal anti-BRD4, Cat# A301-985A50, RRID: AB_2631449. As secondary antibodies horseradish peroxidase-conjugated antibodies HRP-conjugated polyclonal goat anti-mouse IgG H&L, Cat#ab6789; RRID: AB_955439 and HRP-conjugated polyclonal goat anti-rabbit IgG H&L, Cat#ab6721; RRID: AB_955447 from Abcam were used. The quantification of Western blot signals was performed with ImageJ.

### RNA isolation and qRT-PCR

Cells were seeded in 6 well plates and treated after 24 h. After indicated treatment times cells were harvested and total RNA was isolated using the peqGOLD total RNA kit (PeqLab) according to the manufacturer’s instructions. The RNA concentration was determined by NanoDrop and 1 µg of RNA was used for cDNA synthesis using a RevertAid first-strand cDNA synthesis kit (Thermo Fisher Scientific). qRT-PCR was performed using a QuantStudio™ 7 Flex system (Applied Biosystems) with the QuantStudio Design & Analysis software. For the reaction Sybr™ Green PCR mastermix (Applied Biosystems) and the following primes purchased from Eurofins against the human targets were used (Table 1).

**Table 1:** Primers used for qRT-PCR analysis

Target	Forward (5’ ◊ 3’)	Reverse (3’ ◊ 5’)
RPII	GCACCACGTCCAATGACAT	GTGCGGCTGCTTCCATAA
G6PD	ATCGACCACTACCTGGGCAA	TTCTGCATCACGTCCCGGA
c-Myc	CGTCCTCGGATTCTCTGCTC	GCTGGTGCATTTTCGGTTGT
Bcl2a1	GGCAGAAGATGACAGACTGTGAA	TGGTCAACAGTATTGCTTCAGGA
IκBα	GTCAAGGAGCTGCAGGAGAT	ATGGCCAAGTGCAGGAAC
p65	TCAAGAAGAGTCCTTTCAGC	GGATGACGTAAAGGGATAGGG
TNFα	GACAAGCCTGTAGCCCATG	TCTCAGCTCCACGCCATT
Stat3	ACCAGCAGTATAGCCGCTTC	CACAATCCGGGCAATCTCCA
Stat5	GAAAACATATGACCGCTGCCC	CGGAGAGCTGCAATTGTTGG
Pim-1	TCAGGCAGAGGGTCTCTTCA	AGCCAAGGGTGACAGAATCTAC

Relative expression levels of target transcripts were calculated and normalized to the reference transcripts of RPII and G6PD with the ΔΔ*c*_*t*_-method.

### siRNA knockdown

For transient transfection the Neon^®^ transfection system (Thermo Fisher Scientific) and Silencer™ Select siRNAs (Thermo Fisher Scientific) were used. 3 × 10^6^ cells were used per transfection and resuspended in 100 µl T-buffer. Electroporation was performed with 1200 V, 20ms and 2 pulses without addition of siRNA or addition of control siRNA (4390843) or siRNA targeting c-Myc (s1929 sic-Myc #1, s1930 sic-Myc #2) at 100nM prior to electroporation. To increase transfection efficacy, cells were incubated in RPMI-1640 GlutaMAX-I without P/S for 24 h before a second electroporation was performed. At 6, 24 and 48 h after the second electroporation, cell death was measured and samples for qPCR were prepared as previously described. 24 h after the 2nd transfection samples for Western Blot analysis were prepared as described.

Additionally, knockdown of c-Myc using sic-Myc #1 and sic-Myc #2 pooled at 100nM final concentration and Bcl2a1 using siBcl2a1 #2 (s1917) and siBcl2a1 #4 (si1918) pooled at 100nM final concentration was performed with the respective non-targeting control. For the combined knockdown the final concentration of siRNA was 200nM. At 6 h after the second electroporation cell death was measured by Annexin V/PI staining and samples for Western Blot analysis were prepared.

### Statistical analysis

Statistical analysis was performed using GraphPad Prism v10 using 2-way ANOVA followed by post-hoc Dunnett’s test. Asterisks indicating significance levels, i.e.: * - *p* < 0.5, ** - *p* < 0.01, *** - *p* < 0.001. If not stated otherwise, data are depicted as mean + SD.

## Results

### BET inhibitors can reduce the expression of BCL2A1

**Fig. 1:**
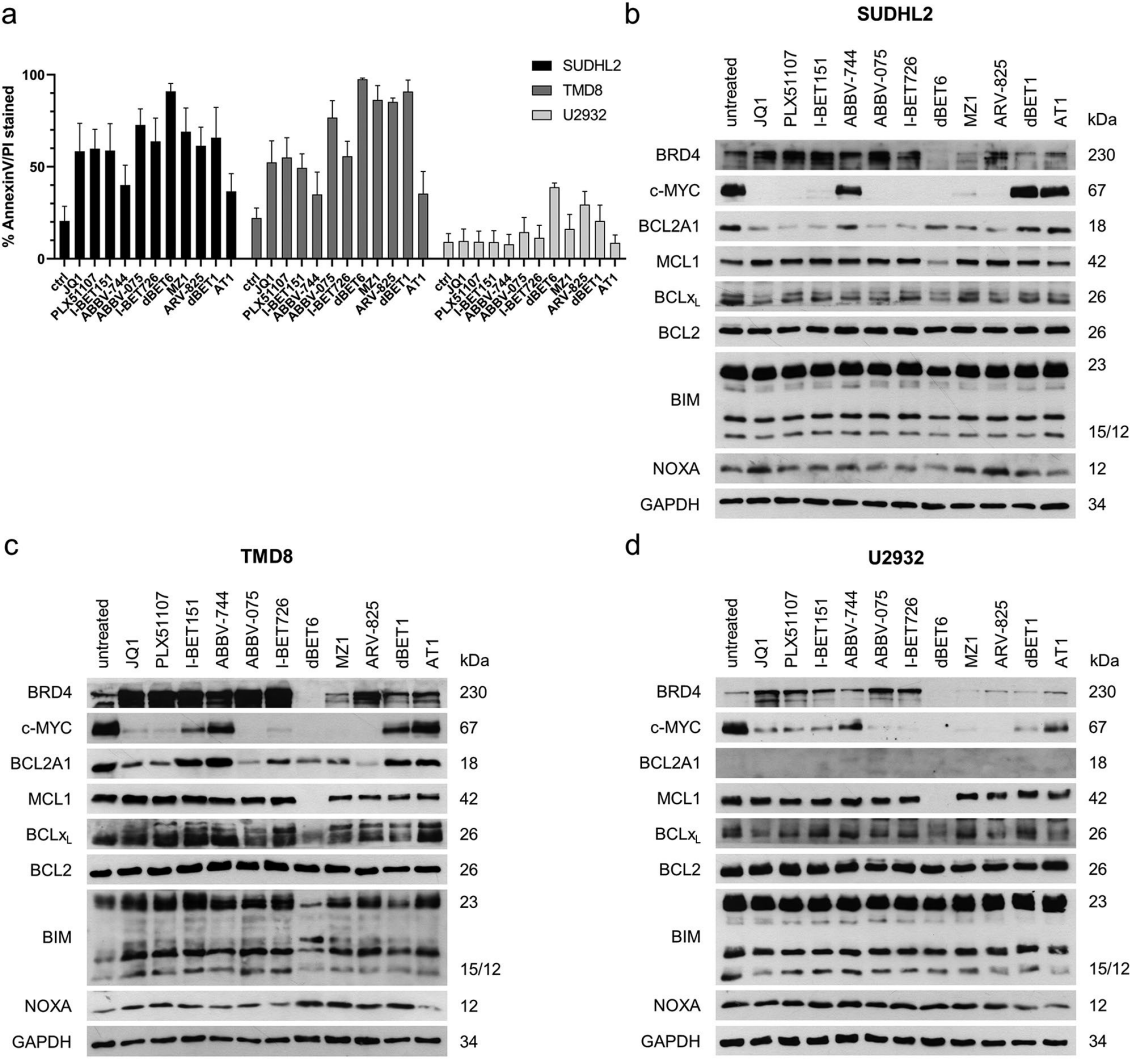
BET inhibitors/-degraders change the protein expression and induce cell death in DLBCL. **a**) Induction of cell death [%] after 24 h treatment with the indicated BET inhibitors/-degraders at 1µM measured by AnnexinV-FITC/PI staining and flow cytometry [data are shown as mean + standard deviation (SD) with *n* = 3]. **b**-**d**) Western Blot of BRD4, c-MYC and BCL2-familiy proteins after 24 h treatment with the indicated BET inhibitors/-degraders at 1µM in **b**) SUDHL2, **c**) TMD8 and **d**) U2932 cells with GAPDH serving as housekeeping control [one representative blot out of three independent experiments is shown]

DLBCL cells display a heterogeneous expression of BCL2A1, which is not associated with the ABC or GCB subtype (Supplementary Fig. 1). In order to reflect the heterogeneous expression of BCL2A1 in DLBCL, three cell lines (SUDHL2, TMD8, U2932) with varying degrees of endogenous BCL2A1 levels were selected to investigate the effect of BET inhibition (BETi) on BCL2A1 levels. Initially, 11 compounds consisting of both BET inhibitors and BET degraders were screened at a concentration of 1 µM, to determine their ability to induce cell death in DLBCL cell lines (Fig. 1a). All compounds were able to induce some extent of cell death in the BCL2A1 expressing SUDHL2 and TMD8 cells, whereas the U2932 cells with low BCL2A1 expression were generally less sensitive.

Since BETi are known to alter the expression of the BCL2 protein family [32, 33], the effects on pro- and anti-apoptotic BCL2 proteins were investigated by Western blotting To determine the efficacy of the compounds, the targeted BET protein BRD4 and a well-established BET transcriptional target, c-MYC were analyzed as well. In all three cell lines, the acetyl-lysine competitive inhibitors JQ1, PLX51107, I-BET151, ABBV-744, ABBV-075 and I-BET726 increased the level of BRD4 protein, while the PROTACs dBET6, MZ1, ARV-825, dBET1 and AT1 reduced the amount of BRD4 in all cell lines. The expression of c-MYC showed a significant reduction in most BETi treated cells, except in cells treated with the BD2 (second bromodomain) specific inhibitor ABBV-744, dBET1 and AT1 (Fig. 1b-d). Overall, treatment with BETi led to a reduction in BCL2A1, with the compounds that did not downregulate c-MYC also failing to reduce BCL2A1 protein levels, confirming our hypothesis that BETi treatment may inhibit BCL2A1. Of note, among the BCL2 proteins, BCL2A1 showed the most prominent regulation by BETi with no effects being observed for the related anti-apoptotic proteins BCL2 and MCL1, except for dBET6, which induced a dramatic reduction of MCL-1. In the TMD8 cells, the BH3-only proteins BIM and NOXA showed slightly increased levels, while only some compounds increased NOXA levels in the SUDHL2 cells and none in the U2932 cells.

**Fig. 2:**
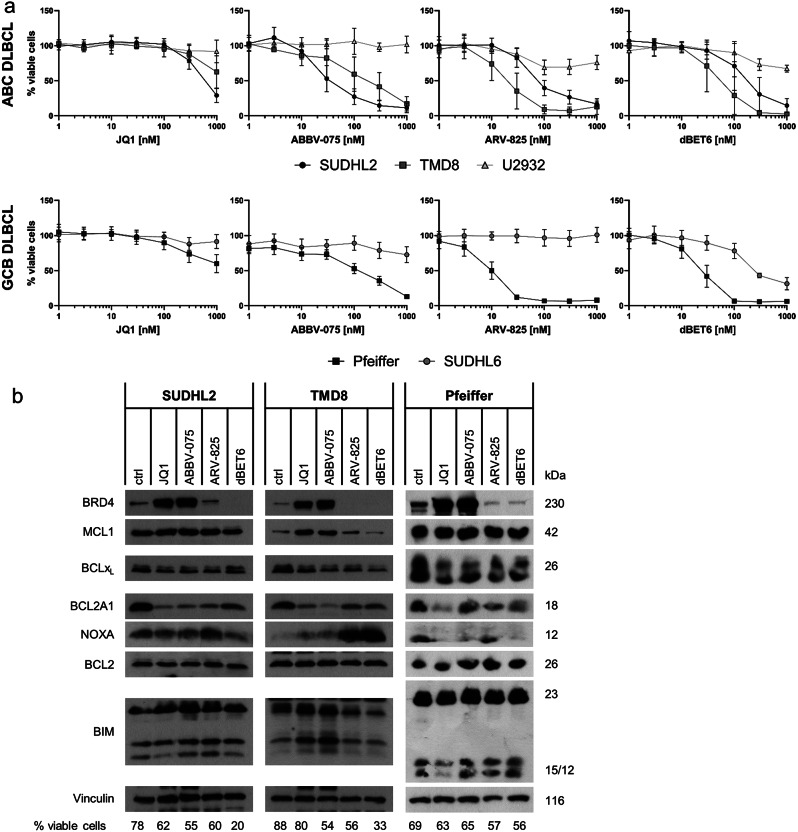
Viability and protein levels are influenced by selected BET inhibitors/-degraders. **a**) Cell viability determined by CTG assay after 48 h treatment with the indicated BET inhibitors/ degraders in ABC and CGB cell lines [data are shown as mean +/- standard deviation (SD) with *n* = 3]. **b**) Western Blot of BRD4 and BCL2 familiy proteins in SUDHL2, TMD8 and Pfeiffer cells treated with JQ1 [1µM], ABBV-075 [SUDHL2 and Pfeiffer 100nM/TMD8 300nM], ARV-825 [SUDHL2 100nM/TMD8 30nM/Pfeiffer 10 nM] and dBET6 [SUDHL2 100nM/TMD8 30nM/Pfeiffer 10 nM]. Cell viability determined by AnnexinV-FITC/PI staining and flow cytometry after 24 h incubation is indicated below the Western blot [one representative blot out of three independent experiments is shown]

Of the 11 initial compounds, JQ1, ABBV-075, ARV-825 and dBET6 showed robust effects on BRD4, c-MYC and BCL2A1 and thus were selected for further studies. To investigate the cytotoxicity of these compounds in more detail, a range of concentrations was investigated by CellTiter-Glo viability assay in both ABC and GCB cell lines (Fig. 2a). Metabolic activity was used as a read-out since some BETi are described to exert cytostatic rather than cytotoxic effects [32]. Overall, the U2932 and SUDHL6 cells were less responsive than the TMD8, Pfeiffer and SUDHL2 cells. Interestingly, the more resistant cells U2932 and SUDHL6 showed low expression of BCL2A1, while the sensitive cells all express high BCL2A1 (Supplementary Fig. 1). Of the cell lines tested, SUDHL2 cells were most sensitive to the two inhibitors (JQ1 and ABBV-075), whereas the Pfeiffer cells responded best to both degraders (ARV-825 and dBET6).

Next, the changes in protein expression were investigated in the sensitive cell lines (Fig. 2b). For these experiments, concentrations of the BET inhibitors were chosen that induced moderate effects around the respective IC50 concentration based on the dose-response curves. All compounds reduced BCL2A1 expression levels and interestingly, the BET inhibitors JQ1 and ABBV-075 seem to be more effective in reducing BCL2A1 expression than the selected BET degraders ARV-825 and dBET6. BET inhibitor treatment coincided with increases in MCL1 and BIM protein in the TMD8 cells but not in the SUDHL2 or Pfeiffer cells.

**Fig. 3:**
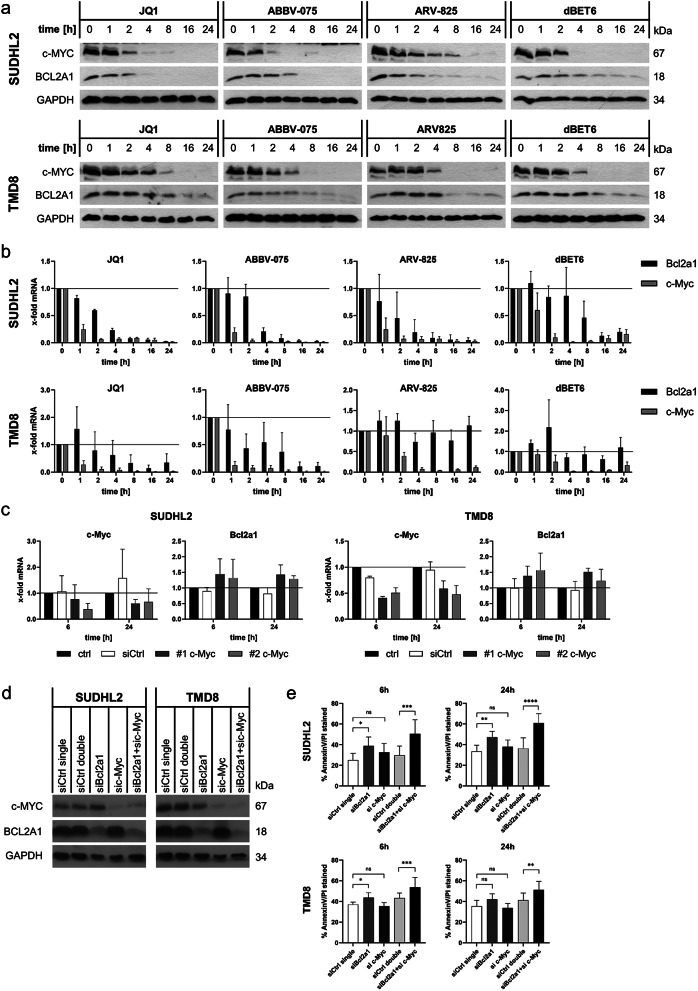
c-Myc and BCL2A1 synergize to prevent cell death. **a**) Protein expression of c-MYC and BCL2A1 in SUDHL2 and TMD8 cells upon treatment with JQ1 [1µM], ABBV-075 [SUDHL2 100nM/TMD8 300nM], ARV-825 [SUDHL2 100nM/TMD8 30nM] or dBET6 [SUDHL2 100nM/TMD8 30nM] for up to 24 h. GAPDH serves as loading control and one representative blot out of three independent experiments is shown. **b**) mRNA expression of c-Myc and Bcl2a1 was assessed by qRT-PCR in the same experiments. Data are shown as mean + standard deviation (SD) with *n* = 3. **c**) Silencing of c-MYC was performed using two individual siRNAs (#1 and #2) followed by analysis of c-Myc and Bcl2a1 mRNA expression at 6 and 24 h after 2nd electroporation. **d**-**e**) siRNA-mediated knockdown of Bcl2a1 and c-Myc alone and combined in SUDHL2 and TMD8 cells was performed with two pooled siRNA sequences for each target. **d**) Knockdown efficiency was confirmed by Western Blot 24 h after the 2nd electroporation. **e**) Viability of electroporated cells 6 and 24 h after the 2nd electroporation measured by AnnexinV-FITC/PI and flow cytometry [data are shown as mean + standard deviation (SD) with *n* = 3]. For individual and combined knockdown, the concentration of non-targeting siCtrl was adapted to match the concentration of targeting siRNA

Since the expression levels of c-MYC and BCL2A1 were found to correlate in the initial screen (Fig. 1b-d), we hypothesized that BCL2A1 and c-MYC may be regulated by the same mechanisms or even through a direct interaction. Therefore, kinetics experiments were performed in the SUDHL2 and TMD8 cells by Western blotting and qPCR monitoring time points up to 24 h treatment using the selected BETi (Fig. 3a and b). Effects on BRD4 protein levels were already observed after 1 h of treatment, with cell death starting at 8 h (Supplementary Fig. 2). An almost complete loss of c-MYC was observed after 4 h treatment (Fig. 3a), while mRNA expression was markedly downregulated already after 1 h (Fig. 3b). BCL2A1 protein levels started to decrease after 4 h treatment and continued to drop up to 24 h. Both methods reveal a decrease of c-MYC levels preceding the BCL2A1 reduction. Overall, the response to BET inhibition appeared to be faster in the SUDHL2 than in the TMD8 cells, indicating that cell intrinsic factors may regulate the response to BET inhibitors. Generally, the reduction of c-Myc mRNA was even faster upon treatment with BET inhibitors compared to BET degraders and also the decrease of Bcl2a1 mRNA was more efficiently induced by the inhibitors.

### BCL2A1 and c-MYC synergize to prevent cell death

Given that the effect of BETi treatment on c-MYC was faster than the effects on BCL2A1, we asked whether BCL2A1 may be a downstream transcriptional target of c-MYC and hence indirectly regulated, as suggested by a previous report [34]. To investigate this hypothesis, an siRNA mediated knockdown of c-Myc was performed in the SUDHL2 and TMD8 cell lines (Fig. 3c and Supplementary Fig. 3a). Of note, knockdown of c-Myc did not reduce the cell viability over 48 h, although c-MYC protein levels were successfully decreased (Supplementary Fig. 3a-b). However, BCL2A1 levels were not attenuated in response to the knockdown on either protein or mRNA level (Fig. 3c and Supplementary Fig. 3a). These data indicate that Bcl2a1 is unlikely to be a direct target of c-MYC and that the downregulation of BCL2A1 by BETi treatment is not due to a direct regulation by c-MYC. To investigate which of these players are important to ensure cellular survival, we next performed individual and combined knockdown of BCL2A1 and c-MYC (Fig. 3d-e). While loss of c-MYC again did not induce cell death, loss of BCL2A1 on its own was sufficient to induce significant cell death in both SUDHL2 and TMD8 cells. Combined knockdown of BCL2A1 and c-MYC further increased cell death, indicating that both proteins synergize to maintain viability (Fig. 3e).

### BET inhibitors influence canonical NFκB activity and STAT signaling

**Fig. 4:**
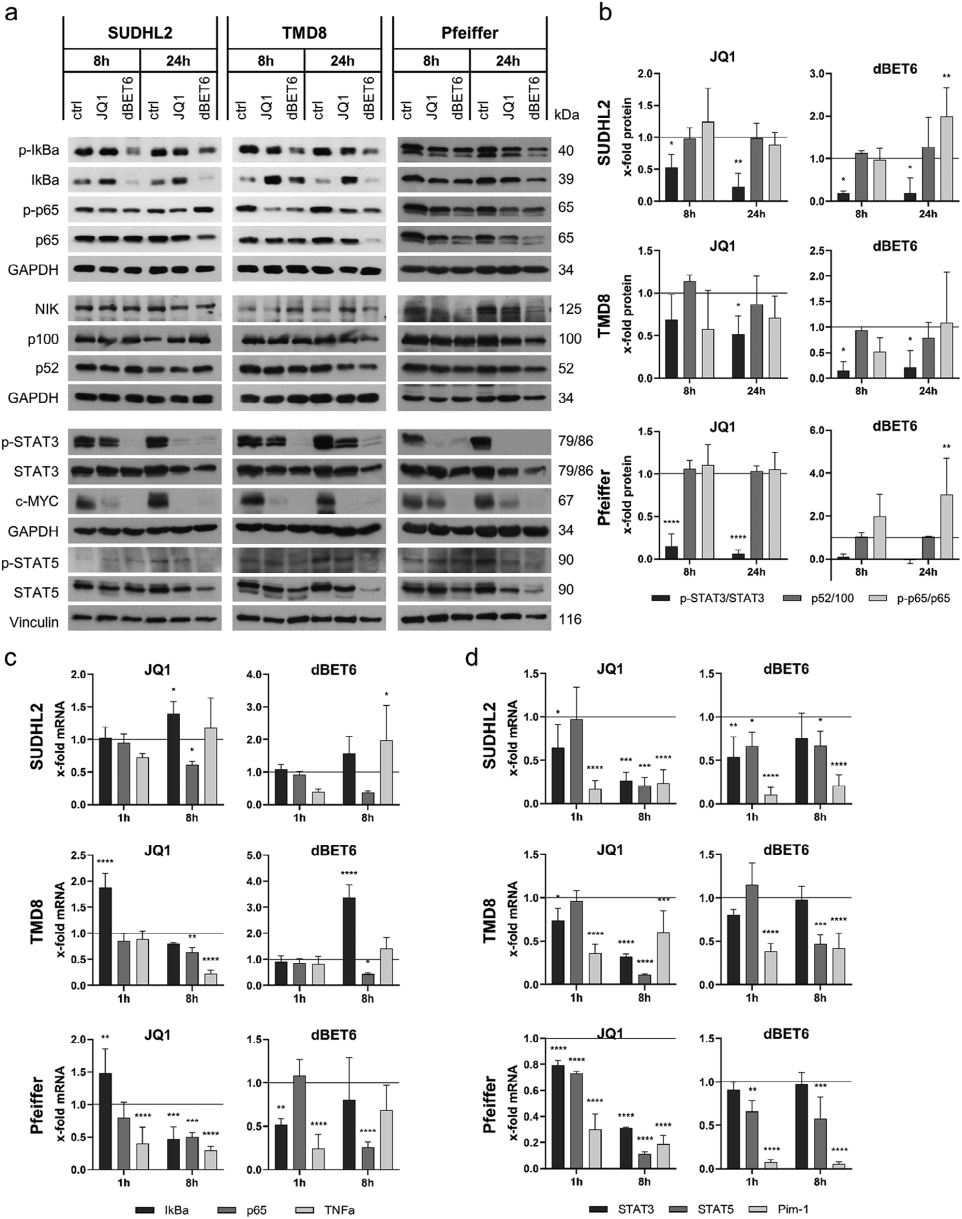
BET inhibitors/-degraders downregulate STAT3 and canonical NFκB signaling. **a**) Western Blot of SUDHL2, TMD8 and Pfeiffer cells treated with JQ1 [1µM] or dBET6 [SUDHL2 100nM/TMD8 and Pfeiffer 30nM] for 8 h and 24 h [one representative blot out of three independent experiments is shown]. **b**) Ratio of phosphorylated to total protein, determined by quantification of Western Blot bands. **c**) mRNA levels of IκBα, p65 and TNFα in SUDHL2, TMD8 and Pfeiffer cells treated with JQ1 [1µM] or dBET6 [SUDHL2 100nM/TMD8 and Pfeiffer 30nM] for 1 h and 8 h. **d**) mRNA levels of Stat3, Stat5 and Pim-1 in SUDHL2, TMD8 and Pfeiffer cells treated with JQ1 [1µM] or dBET6 [SUDHL2 100nM/TMD8 and Pfeiffer 30nM] for 1 h and 8 h [data are shown as mean + standard deviation (SD) with *n* = 3]

Next, we asked how expression of BCL2A1 is regulated upon BET inhibition. BCL2A1 is described as a target gene of NFκB signaling [35], and since BET inhibition may also regulate NFkB, we investigated how the NFκB pathway was affected by BETi treatment (Fig. 4). The cell lines investigated so far belong to the ABC subtype of DLBCL, which is characterized by a constitutive activation of the NFκB pathway [36], as demonstrated here by high endogenous phosphorylation of IκBα and p65 (Fig. 4a). ABC DLBCL also often harbors mutations in the CARM1, BCL-10, MALT1 complex that lead to increased IKK activation, whereas the GCB subtype rather displays an activated c-Rel profile often induced by gene amplifications [26, 37]. To include both subtypes in our analysis, we extended our study to the GCB cell line Pfeiffer that also expresses high levels of BCL2A1 comparable to SUDHL2 (Supplementary Fig. 1).

Analysis of the canonical pathway showed a modest reduction of p-IκBα upon JQ1 treatment, indicating reduced activation of the canonical pathway (Fig. 4a). Interestingly, JQ1 induced an increase of IκBα protein in the ABC DLBCL cell lines, which was very pronounced especially in the TMD8 cells, where mRNA levels were also transiently increased (Fig. 4c). In line with reduced activation of the canonical pathway, the levels of phosphorylated p65 were also attenuated upon JQ1 treatment in all cell lines (Fig. 4b). Treatment with dBET6 resulted in a strong reduction of phosphorylated and total IκBα protein levels. Furthermore, p65 protein levels were strongly decreased upon prolonged incubation times. This loss of p65 was transcriptionally regulated, as seen in the prominent reduction of p65 mRNA after 8 h treatment with dBET6 (Fig. 4c).

Since the phosphorylation of Ser536 of p65 has been suggested to regulate the translocation of this protein into the nucleus and therefore its transcriptional activity [38], immunofluorescence staining of p65 was performed to investigate whether the amount of p65 in the nucleus differed upon BETi treatment. Treatment with JQ1 and dBET6 both induced a slight decrease of p65 signal intensity, however this happened in both the nuclear and the cytosolic region. This indicated that the rate of translocation of p65 stayed unaffected and there was a downregulation of the general pool of p65 in the cell rather than a changed localization as reaction to BETi treatment (Supplementary Fig. 4). This tendency of overall p65 reduction was also observed in Western Blot analysis of fractionated lysates (Supplementary Fig. 4).

To assess the effect of BETi on the non-canonical NFκB pathway, we investigated the protein levels of p100 and p52, as well as the expression of NIK (Fig. 4a and b). Treatment with either JQ1 or dBET6 did not alter the processing of p100 to p52, indicating that the non-canonical pathway was not strongly affected by BETi treatment. In the Pfeiffer cells, a reduction of NIK was observed upon BETi treatment that was not detected in TMD8 or SUDHL2 cells.

Many ABC DLBCL cell lines present constitutive activation of the JAK/STAT signaling axis [39]. Since c-MYC may also be influenced by STAT signaling and since STAT3 has also been described as BET-regulated transcription factor, we next analyzed activation of STAT signaling [30, 40, 41]. Regardless of the subtype, treatment with JQ1 and dBET6 resulted in significantly decreased levels of phosphorylated STAT3 protein after 8 and 24 h, which was more pronounced for dBET6 (Fig. 4a and b). Meanwhile only a minor reduction of overall STAT3 protein at longer treatment times was observed in all three cell lines, indicating reduced activation of STAT3 rather than transcriptional regulation (Fig. 4a). On a transcriptional level, Stat3 and also Stat5 mRNA was significantly downregulated by JQ1, but less by dBET6 after 8 h of treatment in all three cell lines (Fig. 4d). The reduced Stat5 mRNA expression was also translated to decreased STAT5 protein levels with prolonged treatment times. Additionally, the mRNA expression of the Pim-1 kinase, a downstream target of STAT signaling [42], was significantly reduced already after 1 h of BETi treatment in all three cell lines.

### TPCA-1 treatment influences NFκB and STAT3 signaling

**Fig. 5:**
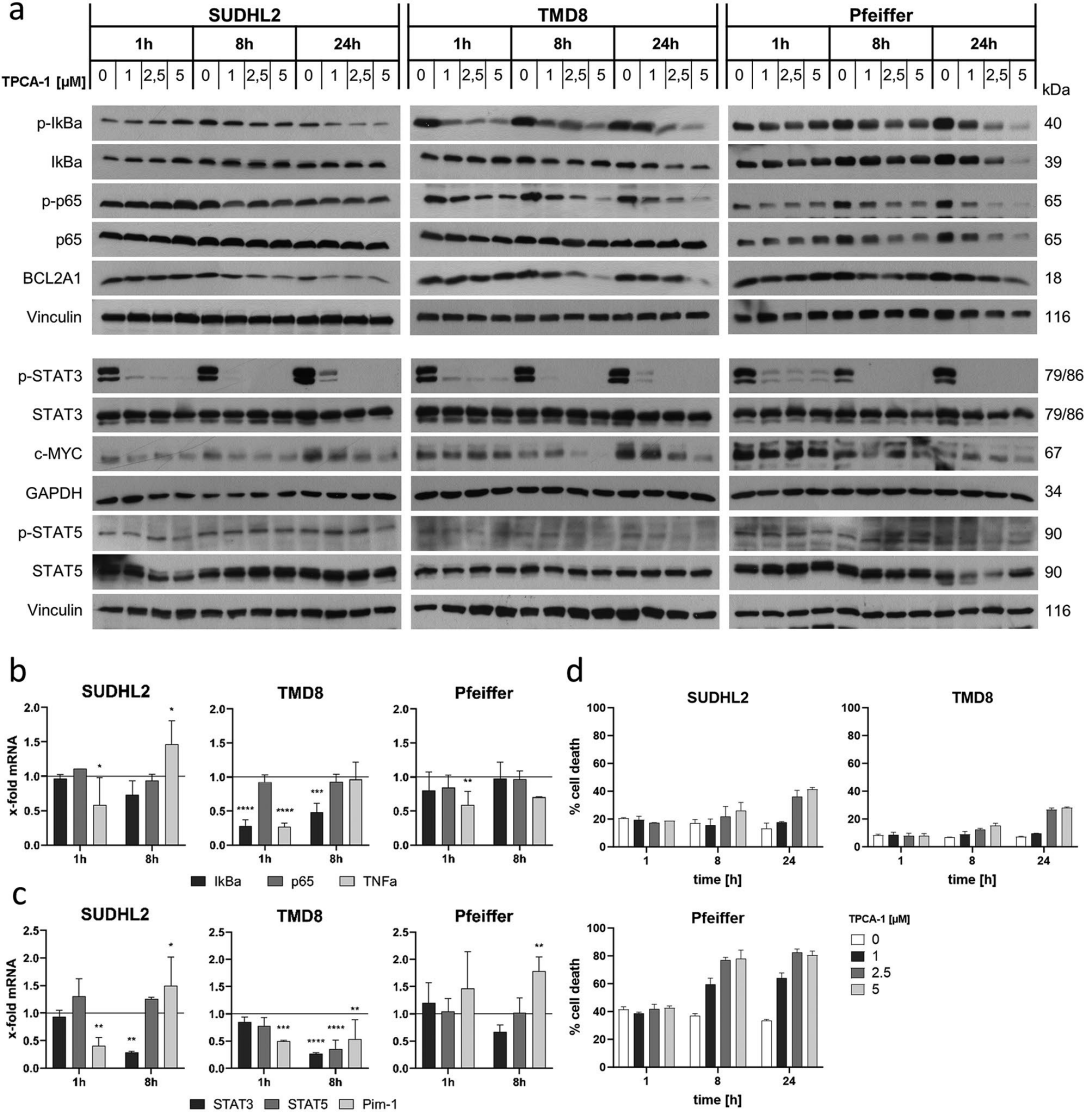
BCL2A1 protein levels and STAT3 phosphorylation are downregulated upon TPCA-1 treatment in SUDHL2, TMD8 and Pfeiffer cells. **a**) Western Blot of SUDHL2, TMD8 and Pfeiffer cells after 1, 8 and 24 h of TPCA-1 treatment with increasing concentrations [one representative blot out of three independent experiments is shown]. **b**) mRNA expression of IκBα, p65 and TNFα and **c**) mRNA expression of Stat3, Stat5 and Pim-1 after 1 and 8 h of TPCA-1 [1µM] treatment [data are shown as mean + standard deviation (SD) with *n* = 3]. **d**) Cell death induction upon TPCA-1 treatment for 1, 8 and 24 h determined by FACS FSC/SSC [data are shown as mean + standard deviation (SD) with *n* = 3]

To further characterize the role of NFκB in the regulation of BCL2A1, the pan-IKK inhibitor TPCA-1 was used. Inhibition of the NFκB pathway was confirmed by Western blotting showing changes in the phosphorylation of IκBα and p65 already after 1 h treatment in the TMD8 and Pfeiffer cells. Interestingly, in the SUDHL2 cells, NFκB signaling was not affected by TPCA-1 treatment, and also mRNA expression of p65 or IkBα was not altered in these cells (Fig. 5a and b). Besides its intended effect on IKKs, TPCA-1 has been also described as inhibitor of STAT3 [43]. Indeed, STAT3 phosphorylation was already inhibited by TPCA-1 within 1 h, leading to a complete loss of STAT3 phosphorylation after 8 h of treatment in all cell lines (Fig. 5a). The loss of STAT3 phosphorylation was not associated with changes in total STAT3 levels and also the activation or expression of STAT5 was not affected (Fig. 5a and c). Of note, TPCA-1 induced a dose-dependent decrease of BCL2A1 protein after 8 h and 24 h of treatment in all three cell lines. In addition, also c-MYC protein was reduced by TPCA-1 at 8 and 24 h of treatment. Treatment with TPCA-1 resulted in moderate cell death induction at prolonged treatment times and higher concentrations in all cell lines investigated (Fig. 5).

Taken together, these studies highlight that TPCA-1 treatment and BET inhibition both resulted in significantly altered STAT3 and NFκB signaling that was accompanied with loss of BCL2A1 and c-MYC expression (Graphical abstract). However, both compound classes had different transcriptional effects especially regarding the transcription of p65, which was reduced by BETi but not by TPCA-1. Therefore, we conclude that BET proteins may act as master regulators of both NFκB and STAT signaling, and that both of these pathways may contribute to a high expression of c-MYC and BCL2A1 in DLBCL. Thereby, our study unraveled a new complex regulatory network in DLBCL that can be addressed by BET inhibition to reduce key oncogenic drivers and induce cell death.

## Discussion

The inhibition of cell death in cancer cells through the deregulation of apoptotic signaling is a common strategy employed by cancer cells. Hematological malignancies often show increased expression of anti-apoptotic proteins, such as MCL1 or BCL2A1 that protect the cells from apoptosis induction. In particular DLBCL displays a high but very heterogeneous expression of the anti-apoptotic protein BCL2A1, indicating a potential role of BCL2A1 in tumourigenesis and giving rise to our hypothesis that BCL2A1 may be a promising therapeutic target. However, a clinically used BH3-mimetic inhibiting BCL2A1 has not yet been identified. Therefore, we hypothesized that indirect inhibition of BCL2A1 on an epigenetic level may reduce its expression and investigated an initial set of 11 compounds comprising classical inhibitors and specific degraders of BET proteins.

The BETi showed differential efficacy in inducing cell death in five DLBCL cell lines with different endogenous BCL2A1 expression levels, with the BET degraders overall being more potent in inducing cell death than the acetyl-lysine inhibitors. In this regard, the first generation BET inhibitor JQ1 was less potent in inducing cell death than second generation BETi, which may reflect the improved BET targeting efficacy [44]. In contrast, the BET degraders ARV-825 and dBET6 both prompted strong induction of cell death, highlighting the potential of using the specific targeting of PROTAC molecules to increase apoptosis of cancer cells with lower drug concentrations and potentially less compensatory feedback effects [45]. The efficacy of the BET degraders was demonstrated by the loss of their direct target protein BRD4, while the BET inhibitors caused an accumulation of BRD4, which has been observed previously [46] and may be rooted in the increased protein stability mediated through prolonged half-life of the deubiquitinase DUB3, which protects BRD4 from degradation [47].

Detailed dose titration experiments also highlighted that the sensitivity for the different BETi was to some extent cell type specific as the sensitive cells showed somewhat different response patterns towards the different compounds. The results of clinical studies with single treatment BETi highlight the need for biomarkers to estimate patient responses and interestingly, the basal expression of BCL2 family proteins may act as indicator for response to BETi treatment [48, 49]. The data presented in this study might hint toward a relation between BCL2A1 protein expression and response to BET inhibition. This is in line with our hypothesis that BETi may reduce BCL2A1 expression, thus representing a strategy to indirectly inhibit BCL2A1 and prime the cells for apoptosis. Throughout our study we observed an effect of BETi on BCL2A1 expression both on mRNA as well as on protein level. Thereby, the inhibitors appeared to have a stronger effect on the expression of BCL2A1 than the PROTACs, highlighting the difference between inhibition of the bromodomains of BET proteins and degradation of the multidomain protein BRD4 in regards to secondary effects and interactions through the extra-terminal domain [21].

The balance of pro- and anti-apoptotic proteins determines whether permeabilization of the mitochondrial membrane and apoptosis induction takes place [50]. The hydrophobic residues in the BH3 binding groove of anti-apoptotic proteins define the affinity to BH3-only proteins, allowing for specific or overlapping interactions between proteins. The ability of BH3-only proteins such as BIM and PUMA to bind to all anti-apoptotic proteins enables the expression of one anti-apoptotic protein to compensate for the loss of another [51]. These compensatory effects in the BCL2 protein family are a common response to treatments that induce apoptosis by targeting one BCL2 protein, hence the protein levels of the prominent members of the family were included in our investigations.

Previous studies have shown that BETi influence the expression of BCL2 family proteins, with an upregulation of BIM and downregulation of the anti-apoptotic BCL2 and BCLx_L_ being commonly described [21, 32, 52]. In this study, we only observed only minor effects on all BCL2 proteins apart from BCL2A1. Treatment with BETi can result in a global reduction of transcription through reduced occupancy of BRD4 at promoter sites [53], which may provide an explanation for the pronounced effects on BCL2A1 as very short-lived protein [54, 55].

Apart from BCL2A1, we consistently observed prominent reduction of c-MYC, which is also a short-lived protein. Inhibition of BET proteins reduces their occupancy at the c-Myc promoter and leads to subsequent reduction of c-Myc transcription [56, 57]. The decreased transcription of c-Myc and Bcl2a1 was followed by a fast reduction of both short-lived proteins. BCL2A1 and c-MYC are both described to be relevant for cancer cell survival and additionally seem to correlate in a c-MYC overexpression context [34, 58], indicating similar regulation of both genes and shared upstream signaling pathways. Our study also indicates that loss of c-MYC increases the anti-apoptotic function of BCL2A1, and that combined inhibition of BCL2A1 and c-MYC is sufficient to induce apoptosis in DLBCL.

The transcription of BCL2A1 is thought to be mainly regulated through NFκB signaling, and therefore we initially aimed to characterize the effects of BETi on NFκB signaling. While the non-canonical signaling pathway was largely unaffected by BETi, we observed a reduction of p-IκBα accompanied by an increase in IκBα upon JQ1 treatment as also observed by Ceribelli et al. [59], indicating less activation of the IKK complex. However, additional effects of BET inhibition might be due to non-canonical interactions of BET proteins with acetylated proteins in the cell. BRD4 may for example bind to acetylated p65 directly, an interaction that is suppressed upon JQ1 treatment [60].

Since the BCL2A1 promoter region displays a BRD4 binding site [61], we further wanted to distinguish between direct effects of inhibition of BRD4 and the effects of NFκB signaling in regulation of BCL2A1 protein upon BET inhibition. To this end, we treated the cells with increasing concentrations of the pan-IKK inhibitor TPCA-1 and observed dose dependent reduction of BCL2A1 protein and cell death induction. In contrast to the treatment with BETi, no reduction of p65 mRNA expression was observed upon TPCA-1 treatment, indicating that inhibition of BET proteins affects the transcription and feedback loops of NFκB molecules differently than a direct IKK inhibitor. Furthermore, it is noteworthy that the SUDHL2 cells display no prominent reduction of canonical NFκB signaling but the strongest reduction of BCL2A1 protein out of the three cell lines. This prominent effect of TPCA-1 on BCL2A1 expression prompted us to also investigate the effects of TPCA-1 on STAT3 signaling, as this may also be influenced by TPCA-1 [43] and the SUDHL2 cells have been described as highly expressing STAT3 [30].

Indeed, TPCA-1 induced a strong and fast reduction of P-STAT3 with a coinciding decrease of c-MYC protein. Taken together, our data indicate that both BETi and TPCA-1 influence STAT3 signaling in addition to NFκB signaling, as partially described previously [59, 60], and that both pathways may influence BCL2A1 and c-MYC expression. Thus, our study indicates a novel regulatory network including STAT3 signaling that may lead to simultaneous regulation of c-MYC and BCL2A1. This conclusion is supported by recent data indicating that ABC DLBCL cases may be segregated into STAT3 high and low expressers which also correlates with a differential expression of BCL2A1 [30]. This might indicate a potential regulation of BCL2A1 in response to STAT3 activation status and might further explain the coinciding effects of BETi on c-MYC and BCL2A1 expression, since BETi downregulate STAT3 signaling which in turn affects c-Myc transcription [40, 62]. An open question remains whether STAT3 also directly regulates BCL2A1. Further research on the relation between STAT3 activation, BCL2A1 levels and response to BETi is needed to determine if any indications for a treatment response can be drawn and translated into the clinic.

In summary, we identify BET inhibitors and degraders that induce cell death and change the expression of apoptosis regulators in DLBCL cell lines. Amongst the BCL2 proteins, we found BCL2A1 to be most affected by BETi treatment, coinciding with a loss of c-MYC. Our data highlight novel strategies to indirectly target BCL2A1 using either BETi or inhibitors of the downstream NFκB and STAT3 pathways. Future studies are warranted to further explore the potential of indirectly targeting BCL2A1 as a therapeutic strategy in DLBCL.

### Electronic supplementary material

Below is the link to the electronic supplementary material.

Supplementary Material 1

Supplementary Material 2

## Data Availability

No datasets were generated or analysed during the current study.
