# Supplementary material for: Inhibition of bromodomain and extra-terminal proteins targets constitutively active NFκB and STAT signaling in lymphoma and influences the expression of the antiapoptotic proteins BCL2A1 and c-MYC
Source: Cell Commun Signal. 2024 Aug 27;22:415. doi: 10.1186/s12964-024-01782-9 (PMC11348570; doi:10.1186/s12964-024-01782-9)

Supplement Methods:

Fractionation of lysates

After harvesting of treated cells as described previously, the cells were resuspended in 200µl mitochondrial isolation buffer (250mM sucrose, 20mM Hepes pH 7.4, 5mM $MgCl_2$, 10mM KCl) with freshly added digitonin (0.02%) and incubated 10min on ice for isolation of membrane and cytosolic fractions. Cells were centrifuged at 1600g for 3min at 4°C and the supernatant was taken as cytosolic fraction. The pellet containing the membrane fractions was lysed as previously described for whole cell lysates.

Immunofluorescence staining

Cells were seeded at 5 x 10^5 c/ml in 1ml RPMI in 6 well plates and treated with JQ1 (1µM) or dBET6 (30nM for Pfeiffer and TMD8, 100nM for SUDHL2) for 2h. Cells were harvested and washed in PBS and resuspended to 1 x 10^6 c/ml in PBS. Next, 100µl suspension per 96 well were seeded in clear bottom, black plates (Greiner Bio-One GmbH) and left to adhere for 30min at RT. Cells were fixed in 4% paraformaldehyde for 15min at RT and subsequently permeabilized with 0.1% Triton X-100 for 15min. After washing 3 times with PBS and blocking with antibody dilution buffer (ADB) (0.9% NaCl, 10mM Tris/HCl pH 7.5, 5mM EDTA, 1 mg/mL BSA) for 30min, the cells were incubated at 4°C over night with mouse anti-p65 antibody, diluted 1:100 in ADB. The next day, after 3 washing steps with PBS the secondary antibody goat anti-mouse IgG (H+L) Alexa Fluor™ 555 (Thermo Fisher Scientific Cat# A-21422, RRID:AB_2535844) diluted 1:500 in ADB and 0.1µg/ml DAPI (in ADB) were added to the cells and incubated for 90min at room temperature. The cells were washed again three times and stored in PBS at 4°C or measured directly at the ImageXpress® Micro XLS Widefield High-Content Analysis System with the MetaXpress® Software (Molecular Devices). Images were analyzed using Cell Profiler for intensity of p65 staining.

Structure of BETi used in this study

(+)-JQ1 (inhibitor)

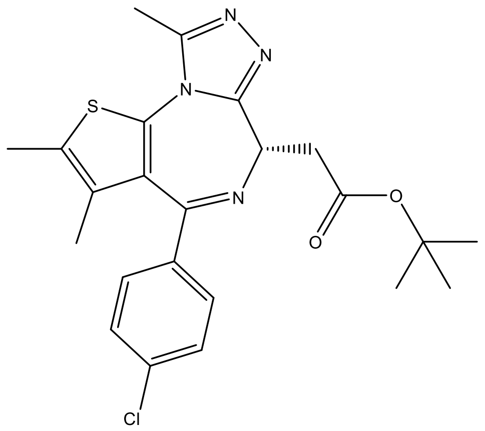

PLX51107 (inhibitor)

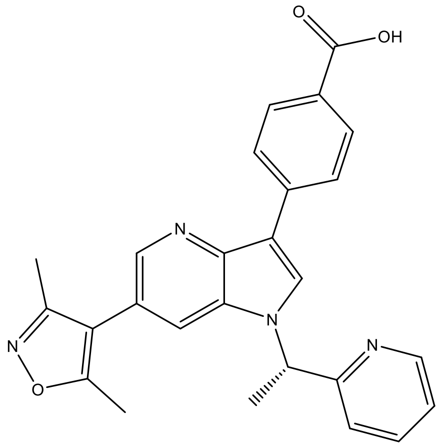

I-BET151 (inhibitor)

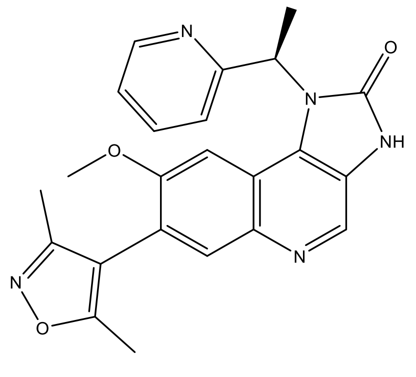

ABBV-744 (inhibitor)

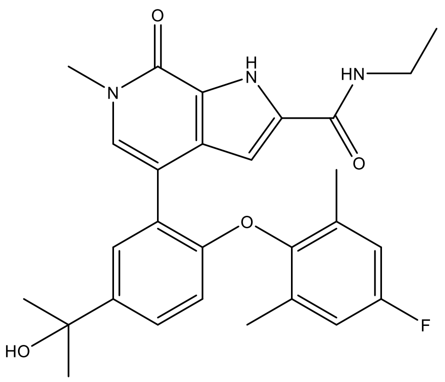

ABBV-075 (inhibitor)

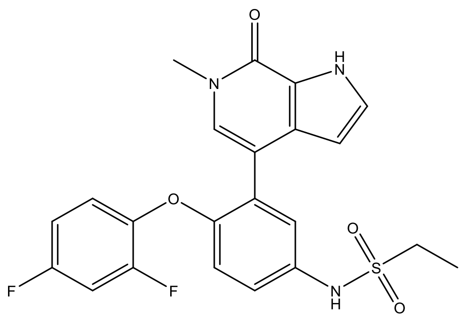

I-BET 726 (inhibitor)

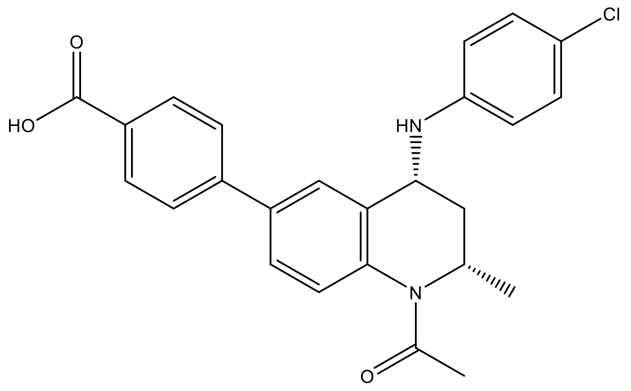

dBET6 (degrader)

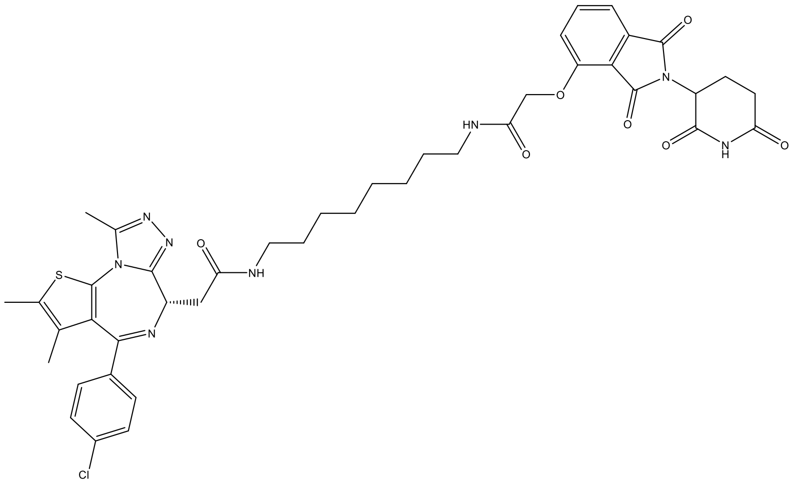

MZ 1 (degrader)

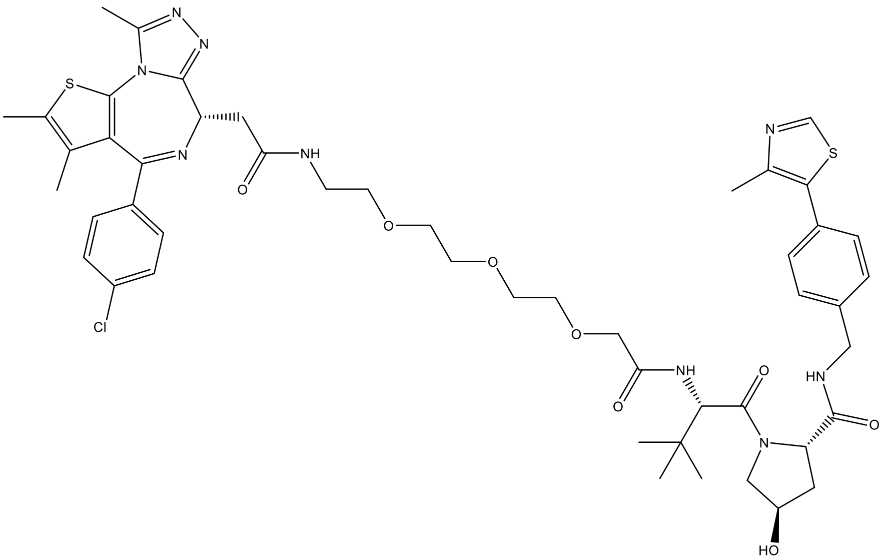

ARV-825 (degrader)

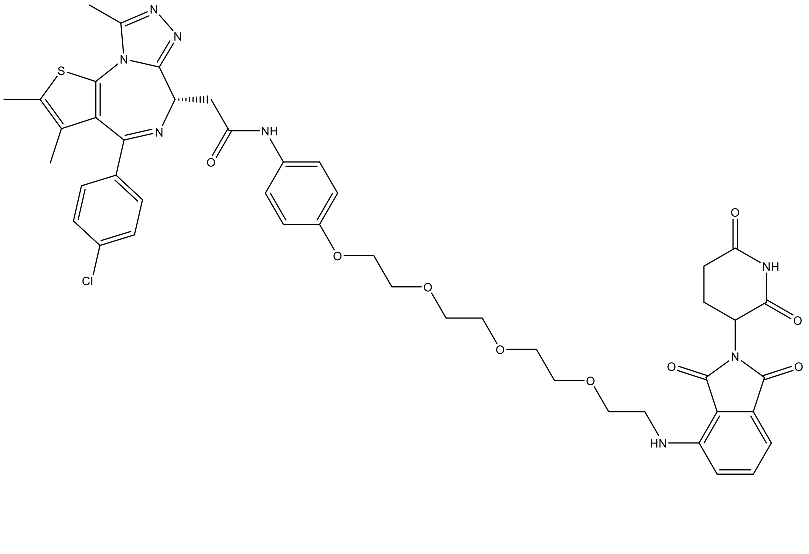

dBET1 (degrader)

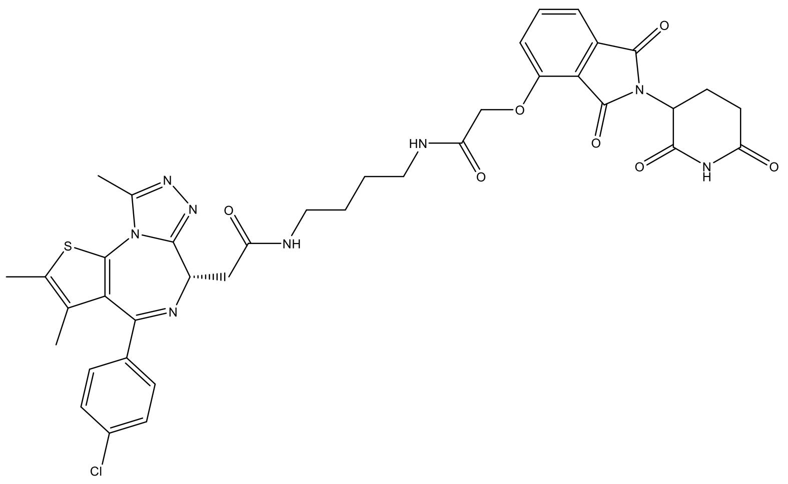

AT1 (degrader)

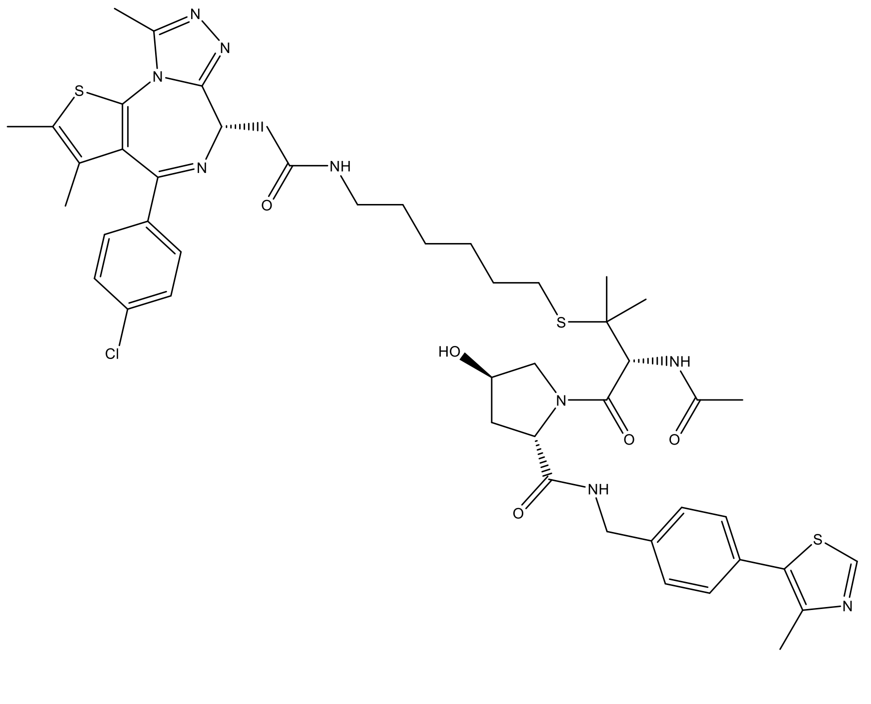

Supplement: Supplementary file 1 — Supplementary Material 1 [file 12964_2024_1782_MOESM1_ESM.pdf]
