# Supplementary material for: Inhibition of bromodomain and extra-terminal proteins targets constitutively active NFκB and STAT signaling in lymphoma and influences the expression of the antiapoptotic proteins BCL2A1 and c-MYC
Source: Cell Commun Signal. 2024 Aug 27;22:415. doi: 10.1186/s12964-024-01782-9 (PMC11348570; doi:10.1186/s12964-024-01782-9)
Supplement: Supplementary file 2 — Supplementary Material 2 [file 12964_2024_1782_MOESM2_ESM.pdf]

Supplementary Figures

Supplementary Figure 1

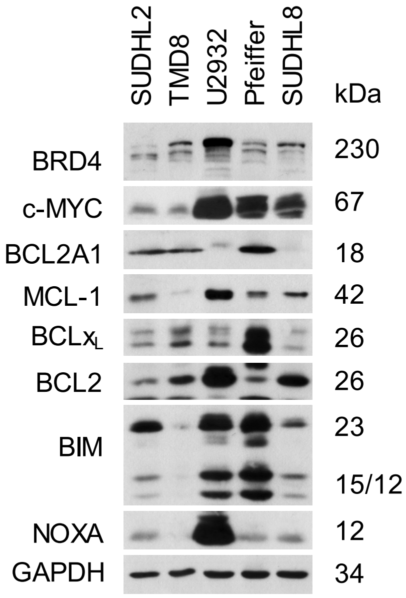

Supplementary Fig. 1: Basal expression of key regulators in DLBCL cell lines (related to Fig. 2)

Western Blot of endogenous protein expression in SUDHL2, TMD8, U2932 (all ABC subtype) , Pfeiffer and SUDHL6 (all GCB subtype) cells [One representative blot out of two independent experiments is shown.].

Supplementary Figure 2

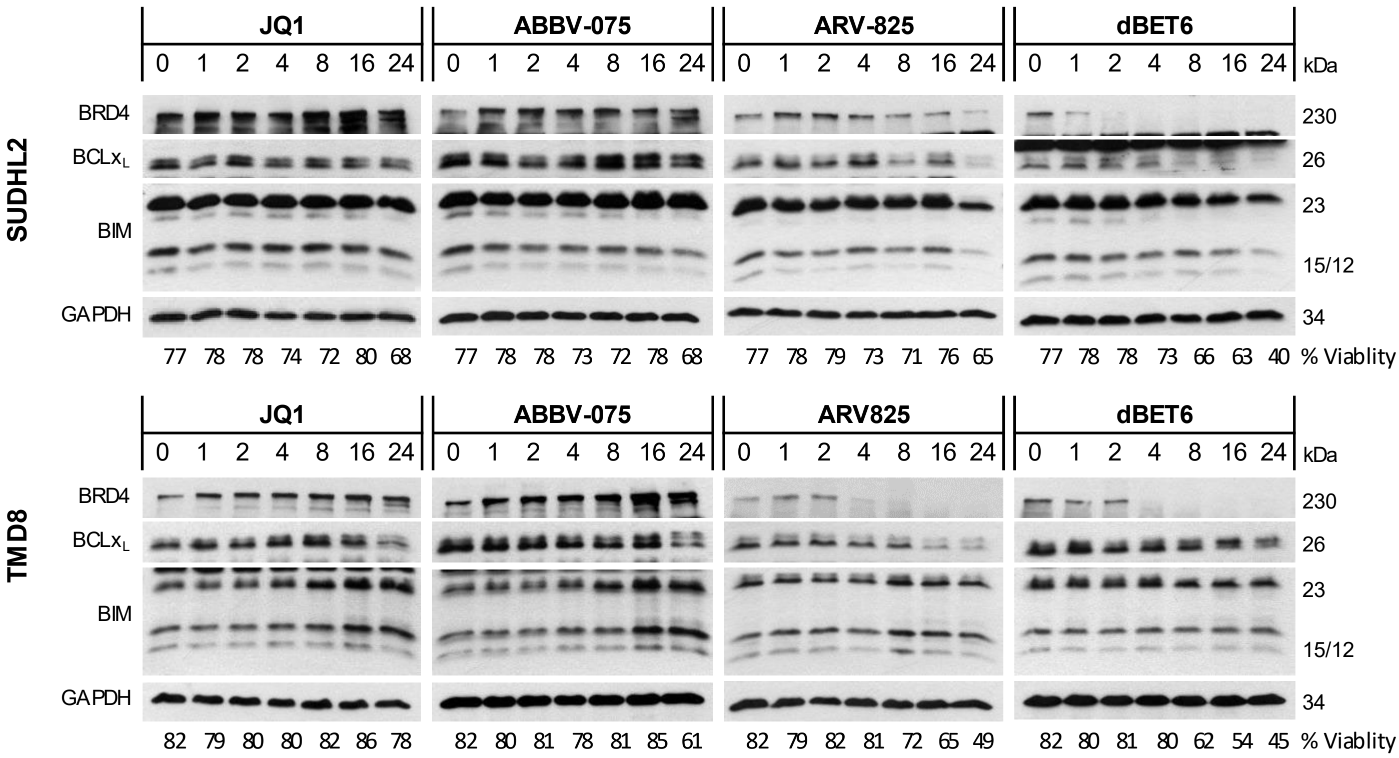

Supplement Fig. 2: Regulation of BRD4 and BCL2 family proteins (related to Fig. 3a)

Western Blot in SUDHL2 and TMD8 cells upon treatment with JQ1 [1µM], ABBV-075 [SUDHL2 100nM/TMD8 300nM], ARV-825 [SUDHL2 100nM/TMD8 30nM] or dBET6 [SUDHL2 100nM/TMD8 30nM] up to 24h. [One representative blot out of three independent experiments is shown.]

Supplementary Figure 3

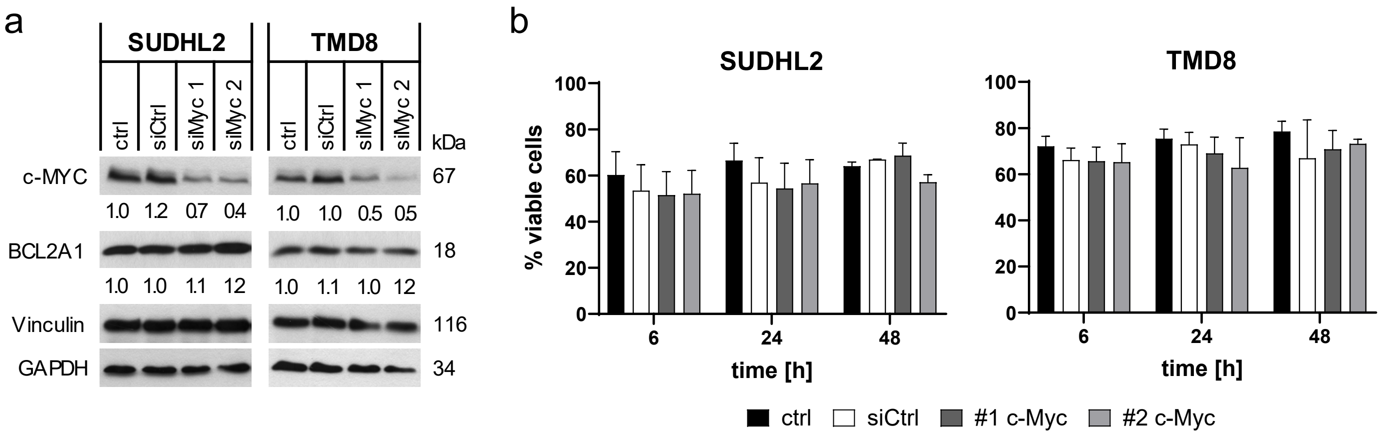

Supplementary Fig. 3: Effects of cMYC knockdown on BCL2A1 protein expression and viability (related to Fig. 5)

a) Western Blot of c-MYC and BCL2A1 expression upon knockdown of c-Myc by two individual siRNA oligonucleotides [One representative blot out of three independent experiments is shown]. Numbers below the blots indicate quantification of normalized protein expression related to the loading controls Vinculin and GAPDH. b) Cell death analysis upon knockdown of c-Myc analysed by staining with AnnexinV-FITC and flow cytometry at 6, 24, and 48 h after knockdown [data shown are mean + SD of thee independent experiments].

Supplementary Figure 4

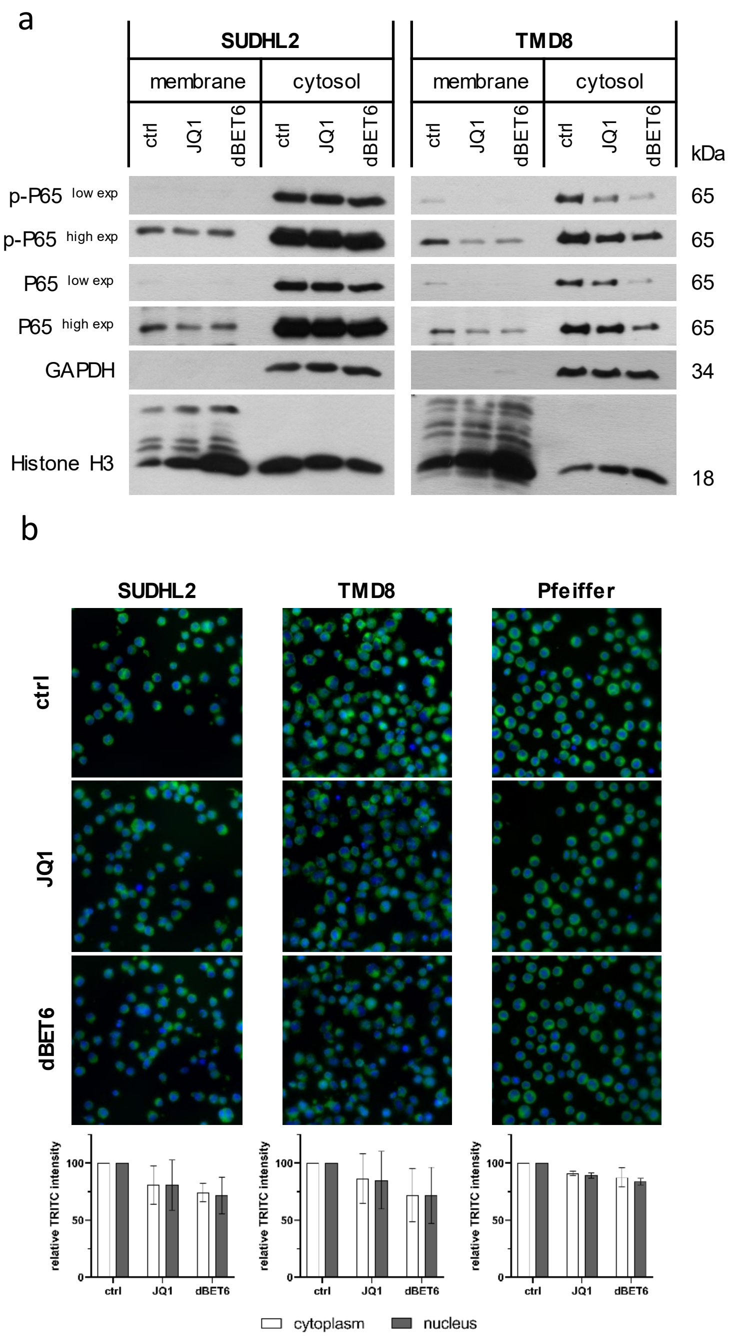

Supplementary Fig. 4: Effects of BETi treatment on p65 translocation (related to Fig. 5)

a) Western blot of fractionated lysates in SUDHL2 and TMD8 cells treated with JQ1 [1µM] or dBET6 [SUDHL2 100nM/TMD8 30nM] for 8h [One representative blot out of three independent experiments is shown.]. b) Immunofluorescence staining of p65 in SUDHL2, TMD8 and Pfeiffer cells treated with JQ1 [1µM] or dBET6 [SUDHL2 100nm/TMD8 30nM] for 2h, Analysis was performed with CellProfiler. [Data are shown as mean + standard deviation (SD) with n=3].
